# Updated practice guideline for dual-energy X-ray absorptiometry (DXA)

**DOI:** 10.1007/s00259-024-06912-6

**Published:** 2024-09-24

**Authors:** Riemer H. J. A. Slart, Marija Punda, Dalal S. Ali, Alberto Bazzocchi, Oliver Bock, Pauline Camacho, John J. Carey, Anita Colquhoun, Juliet Compston, Klaus Engelke, Paola A. Erba, Nicholas C. Harvey, Diane Krueger, Willem F. Lems, E. Michael Lewiecki, Sarah Morgan, Kendall F. Moseley, Christopher O’Brien, Linda Probyn, Yumie Rhee, Bradford Richmond, John T. Schousboe, Christopher Shuhart, Kate A. Ward, Tim Van den Wyngaert, Jules Zhang-Yin, Aliya A. Khan, International Working Group on DXA Best Practices

**Affiliations:** 1 Medical Imaging Centre, Department of Nuclear Medicine & Molecular Imaging (EB50), University Medical Center Groningen, University of Groningen, Hanzeplein 1, PO 9700 RB Groningen, The Netherlands; 2 Department of Oncology and Nuclear Medicine, University Hospital Centre Sestre Milosrdnice, Vinogradska 29, Zagreb, Croatia; 3 Department of Endocrinology, McMaster University, Hamilton, L8S 4L8 Canada; 4 Diagnostic and Interventional Radiology, IRCCS Istituto Ortopedico Rizzoli, Via G. C. Pupilli 1, Bologna, 40136 Italy; 5 Department of Osteoporosis, Inselspital, Bern University Hospital, Switzerland, IG Osteoporose, Bern, Switzerland; 6 Loyola University Medical Center (LUMC), 2160 S 1st Ave, Maywood, IL 60153 USA; 7 University of Galway, Galway, H91 V4AY Ireland; 8 Centre for Osteoporosis & Bone Health, Women’s College Hospital, Toronto, ON Canada; 9 Cambridge Biomedical Centre, Cambridge, UK; 10 Department of Medicine, Institute of Medical Physics, FAU University Erlangen-Nürnberg and Universitätsklinikum Erlangen, Ulmenweg 18, 91054 Erlangen, Germany; 11 Department of Medicine and Surgery, Nuclear Medicine Unit, ASST Ospedale Papa Giovanni, University of Milan-Bicocca, Piazza, Bergamo, Italy; 12 MRC Lifecourse Epidemiology Centre, University of Southampton, Southampton General Hospital, Southampton, UK; 13 NIHR Southampton Biomedical Research Centre, University of Southampton and University Hospital NHS Foundation Trust, Southampton, UK; 14 School of Medicine and Public Health, University of Wisconsin-Madison, Madison, WI USA; 15 Department of Rheumatology, Amsterdam University Medical, Center, The Netherlands; 16 New Mexico Clinical Research & Osteoporosis Center, Albuquerque, NM 87106 USA; 17 The UAB Osteoporosis Prevention and Treatment Clinic, The University of Alabama at Birmingham, Birmingham, Al USA; 18 Division of Endocrinology, Johns Hopkins University, Baltimore, MD 21201 USA; 19 Brant Community Healthcare System, Brantford, ON Canada; 20 Department of Medical Imaging, Sunnybrook Health Sciences Centre, University of Toronto, 2075 Bayview Ave., Toronto, ON M4N 3M5 Canada; 21 Department of Internal Medicine, Endocrine Research Institute, Yonsei University College of Medicine, Seoul, Korea; 22 Diagnostic Radiology, Cleveland Clinic Main Campus, 9500 Euclid Avenue, Cleveland, OH 44195 USA; 23 Park Nicollet Clinic and HealthPartners Institute, Minneapolis, MN USA; 24 Swedish Bone Health and Osteoporosis Center, 1600 E Jefferson St Ste 300, Seattle, WA 98122 USA; 25 Department of Nuclear Medicine, Antwerp University Hospital, Antwerp, Belgium; 26 Department of Nuclear Medicine, Clinique Sud Luxembourg, Vivalia, B-6700 Arlon, Belgium

**Keywords:** Dual-energy X-ray absorptiometry (DXA), Practice guideline, Procedures

## Abstract

The introduction of dual-energy X-ray absorptiometry (DXA) technology in the 1980s revolutionized the diagnosis, management and monitoring of osteoporosis, providing a clinical tool which is now available worldwide. However, DXA measurements are influenced by many technical factors, including the quality control procedures for the instrument, positioning of the patient, and approach to analysis. Reporting of DXA results may be confounded by factors such as selection of reference ranges for T-scores and Z-scores, as well as inadequate knowledge of current standards for interpretation. These points are addressed at length in many international guidelines but are not always easily assimilated by practising clinicians and technicians. Our aim in this report is to identify key elements pertaining to the use of DXA in clinical practice, considering both technical and clinical aspects. Here, we discuss technical aspects of DXA procedures, approaches to interpretation and integration into clinical practice, and the use of non-bone mineral density measurements, such as a vertebral fracture assessment, in clinical risk assessment.

## Introduction

Osteoporosis is a systemic skeletal condition characterized by reduced bone mineral density (BMD) and deterioration of bone microarchitecture leading to bone fragility and increased susceptibility to fracture [1]. The diagnosis of osteoporosis is based on dual-energy X-ray absorptiometry (DXA) measurement of BMD at the lumbar spine, total hip, femoral neck and/or one-third (33%) radius, reported as a T-score using appropriate reference data. Most modern DXA scanners also permit a lateral view of the thoracic and lumbar spine, providing additional information on the presence or absence of vertebral fractures. Best practice recommendations on the use of DXA are summarized in this document providing both technical and clinical recommendations. These recommendations were developed by an International Working Group (IWG) consisting of representatives from International Societies dedicated to enhancing the care of individuals with metabolic bone disease. The recommendations are being published in a series of manuscripts [2]. The following societies have reviewed and endorsed this document: the American Association of Clinical Endocrinology (AACE), the American Society for Bone and Mineral Research (ASBMR), Asian Federation of Osteoporosis Societies (AFOS), Canadian Society of Endocrinology and Metabolism (CSEM), Canadian Association of Nuclear Medicine (CANM), European Association of Nuclear Medicine (EANM), European Calcified Tissue Society (ECTS), European Society for Clinical and Economic Aspects of Osteoporosis, Osteoarthritis and Musculoskeletal Diseases (ESCEO), European Society of Radiology (ESR), European Society of Musculoskeletal Radiology (ESSR), International Osteoporosis Foundation (IOF), International Society for Clinical Densitometry (ISCD), Korean Society of Bone and Mineral Research (KSBMR), and Radiological Society of North America (RSNA).

## Part I: Technical procedures DXA

### Quality control

Both precision and accuracy are necessary in DXA scanning to ensure that the data are clinically meaningful. The precision (reliability) of BMD measurements depends on the technical stability of the instrument, the ability of the technologist to position the patient consistently with repeat scans over time, and the correct analysis of the DXA images [3]. Quality assurance and control procedures vary by manufacturer; some require scanning a calibration block that analyzes, checks and calibrates mechanical function, radiation quality, and absorption coefficient of tissue-equivalent materials [4]. It is also recommended that a spine phantom be scanned daily before use, or at least three times a week [4], with plotting and reviewing of the phantom data. If the results fall outside the acceptable limits and surpass the set thresholds for service, the scanner should be evaluated by a field service engineer.

Each DXA facility should determine its precision error and calculate the least significant change (LSC) for the machine, repeating when a new DXA system is installed [4]. To perform a precision analysis, see the online International Society for Clinical Densitometry (ISCD) 2019 document [4] and the updated version of 2023 [5]. Importantly, precision assessment is not research, but a minimum standard in clinical practice [4, 6, 7].

Cross-calibration procedures are necessary for precise longitudinal assessment when replacing scanners (the same model is usually preferred) or validating measurements between systems (in different institutes). This process is complex and requires detailed knowledge of the process and advanced planning. When replacing a DXA system with another of the same manufacturer and model, cross-calibration should be done by scanning the phantom ten times on each scanner. The measures should be within 1%, but preferably within 0.5% [6]. When replacing an entire system from the same manufacturer but a different technology, or one from a different manufacturer, the LSC using patients representative of the clinic population and sites to be scanned is required on both the old and new systems. For details on how to perform a cross-calibration see the online ISCD 2019 and 2023 documents [4, 5, 8].

### Acquisition techniques and patient preparation and positioning

The patient’s weight and a stadiometer-determined height should be measured at the time of every scan. The limits of body weight range between 120 and 150 kg, depending on different scanner types. A decrease in height (historical ≥ 4 cm or prospective ≥ 2 cm) is an indication for prompt vertebral fracture assessment (VFA, a lateral spine image by DXA) or if not available a conventional lateral radiography of the thoracic and lumbar spine to evaluate for the presence of vertebral fractures [9].

**Fig. 1:**
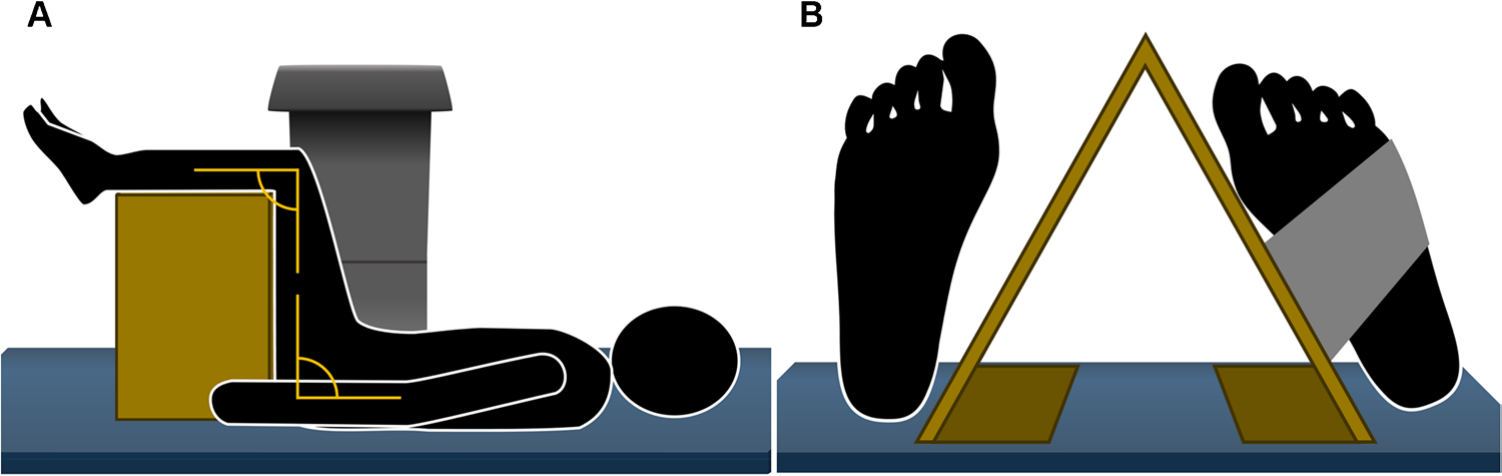
**A** positioning device should be used to ensure that the patient’s hips and knees are flexed to 90° when scanning the lumbar spine. **B** When scanning the hip, the ipsilateral foot should be rotated internally by 15°-20° using a specific positioning device containing a strap for immobilization

A change in weight or fat mass may affect the precision and accuracy of BMD measurements [10]. Variations in position of fat folds (panniculus) can cause nonuniform changes in fat mass distribution therefore, it is recommended that a panniculus is retracted at baseline and follow-up scans. Accuracy in BMD measurement depends on correct patient positioning for every scan performed over time (Fig. 1A). The manufacturer’s specific training and operating recommendations must be followed for best quality, and although procedures are generally the same as presented here, there may be differences. Procedural certification and repeated audits are recommended.

The patient’s body and limbs should be positioned to achieve limited (reduced) effect of tissue thickness on the DXA scan results. Incorrect patient positioning, rotation, or excessive adduction and abduction of the hip and limbs can affect the accuracy of DXA measurements. Although patient-specific physical limitations may affect proper positioning, at the very least, the patient should be consistently positioned for each scan to minimize the effects of rotation, adduction and abduction. The patient should be placed in a flat position, with the limbs and trunk aligned to the body’s midline as closely as possible. Special positioning aids, such as three-sided foam blocks (offering three height options) placed under the knees in the lumbar spine position help to flatten the lumbar lordosis and tangential fan-beam acquisition. A foam cushion or pillow may be used to support the head and neck.

The following points should be considered to ensure accurate patient positioning [7, 8, 11]:

- Lumbar spine

The patient lies supine on the table, legs straight and feet uncrossed. The spine is in a neutral position, with the arms at the sides of the body. The hip and knees are flexed to 90° to reduce the physiological lumbar lordosis and increase the intervertebral spaces and maximize the area of each of the lumbar vertebra (Fig. 1A).

- Hip

In general, there is no significant difference in BMD between both femurs; however, there may be exceptions in some patients who have differential loading of the lower extremities including stroke, prolonged immobility, or other conditions. In routine practice, the non-dominant hip measurement should be taken, rather than either hip, since there may be a difference between them. This is consistent with the NHANES study [12], which used the left side. It is also important to state that the same side should be used for longitudinal measurements. For initial position, the patient’s legs should be straight and parallel, with the toes pointing upwards. The femoral neck should be centered in the scan field, and the lesser trochanter may be visible. Scans should be acquired with a degree of internal rotation of the leg about 15° to 20°, which positions the femoral neck parallel to the scan table plane. Adequate internal rotation should be confirmed with barely visible detection of the lesser trochanter. The appropriate amount of internal rotation can be achieved using a positioning device for the feet, secured with a strap to avoid movement during the acquisition (Fig. 1B) [8]. The leg should be adducted or abducted to be parallel to the edge of the table. It is important to assure that there is no soft tissue included as bone caudal from the femoral neck. If the femoral neck overlaps the ischium, the ischium can be neutralized (removed) from analysis. The femoral neck and total hip region of interest (ROI) positions should be evaluated. The arms of the patient are crossed over the chest to avoid overlapp with a hip area. If one hip is not evaluable, for example in the case of hip arthroplasty, the other side should be assessed. Some guidelines recommend measuring both femurs [13], but in general consensus the non-dominant hip measurement should be taken.

- Forearm

A forearm scan of the non-dominant arm should be considered when the lumbar spine and/or hip scan contain artifacts (e.g. presence of fractures, hip replacements or spine surgical implants or degenerative changes), which limit or impede interpretation, if the patient is over the weight limit of the scanner table, or if the patient cannot mount or be positioned comfortably on the table [3]. A forearm scan should always be performed when possible if the patient has a diagnosis of hyperparathyroidism [4, 14]. If there is a large discordance (more than 1 T-score unit) between the lumbar and hip scans, a forearm scan may be considered. The forearm should be measured and centered with the radius and ulna parallel to the short axis of the scanning table. No hardware, fusion, osteoarthritis, or fractures should be present. A positioner is not used in a Hologic DXA system; for General Electric (GE) systems, a positioner is placed under the forearm for acquisition. Hologic requires that the forearm is measured from the styloid to the elbow and the value is entered into the patient profile, but this is not required for GE. With a GE system, it is important to make sure that the positioner is not identified as “tissue”. In general the patient is in sitting position with the arm on the table, however with the patient in supine position may give less movement artifacts, which are relatively more common in patients sitting by the table [15].

#### Scanning regions of interest

The scan field of a DXA study can include ROIs noted below. The location and size of the ROIs can vary according to the specific scan protocol and purpose of the study (e.g. fracture risk assessment, total body composition).

##### Lumbar spine (Fig. 2A and B)

Hologic Discovery standard has a scan length of 20 cm and a scan width of 11.4 cm. In the analysis the ROI has the same length, but the width is 10.5 cm. The lumbar spine ROI should include the first to fourth lumbar vertebrae. DXA images are generally acquired by the transmission of X-rays from the posterior to anterior direction. They are properly characterized as posterior-anterior (PA) spine scans. Nevertheless, these studies are often called anterior-posterior (AP) spine scans, probably because plain films of the lumbar spine are acquired in the AP projection. Correct and consistent vertebral labeling is essential [8]. The lumbar vertebrae are usually identified by counting from the bottom up, with the iliac crests typically aligned with the L4-L5 intervertebral space. Anatomical variants such as 4 or 6 lumbar vertebrae should be mentioned, if recognisable. The existence of lumbosacral transition vertebrae needs to be considered as a normal anatomic variant and may require a full spine planar radiograph, CT or MRI for correct identification, if specific vertebral labelling verification is needed (e.g. for surgical intervention) and in doubt. Vertebrae affected by local structural changes (e.g. severe osteoarthritic changes, compression fracture, laminectomy) or artifacts (e.g. fusion hardware, vertebroplasty cement, obscured by other implantable devices) are typically excluded. At least two vertebrae should be evaluable for diagnostic classification.

**Fig. 2:**
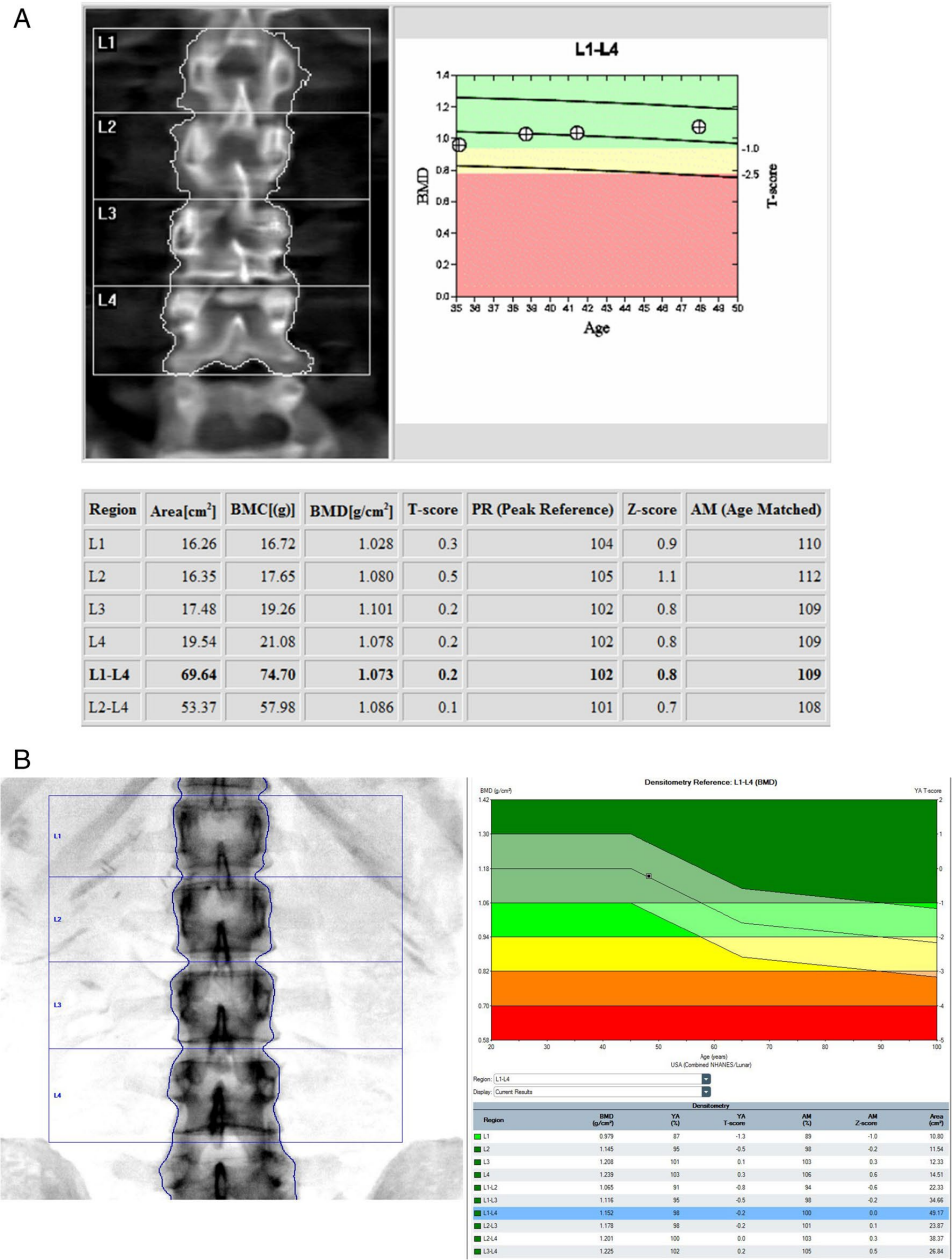
**A** Example Hologic lumbar spine DXA including follow-up. WHO Classification: Normal. **B **Bottom example GE lunar lumbar spine DXA. WHO Classification: Normal

##### Hip (Fig. 3A and B)

 The total hip includes the femoral neck, the proximal femur and the trochanter ROIs. The femoral neck ROI should never include any of the greater trochanteric region. Hologic Discovery standard has a scan length approximately 15 cm and a scan width of 11 cm. In a Hologic scan, the femoral neck ROI is anchored to the top corner of the greater trochanter with a default size of 1.5 × 4.9 cm. The top of the global ROI box is 0.5 cm above the femoral head, 0.5 cm medial of the femoral head, 0.5 cm laterally from the outside edge of the greater trochanter and should extend at least 1 cm below the lesser trochanter. The width of the analysis ROI is about 9 cm but analysis ROI height and width depend on the individual subject’s femur geometry. In a GE scan, the search feature should be used, placing the femoral neck ROI in the narrowest aspect of the femoral neck and the lowest bone mineral content (BMC) with a default width of 1.5 cm. The neck ROI is automatically placed, if incorrect the search feature should be used.

**Fig. 3:**
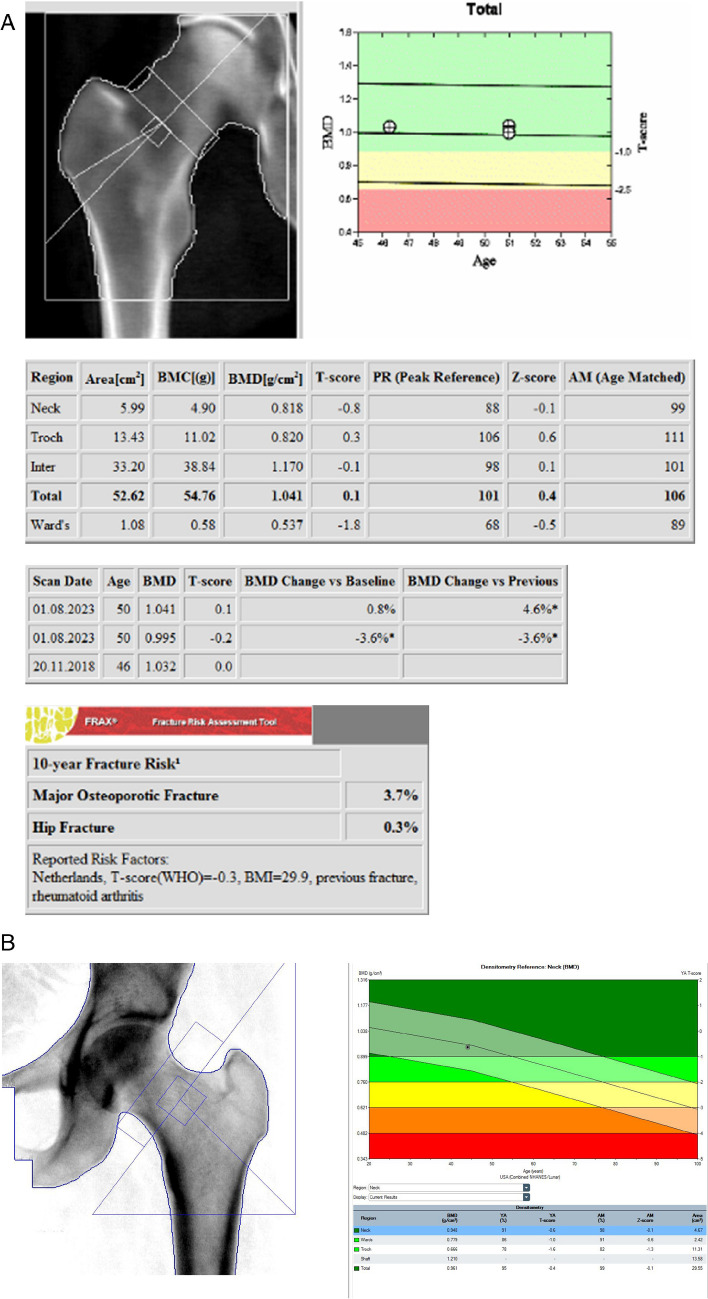
**A** Example Hologic right hip DXA and follow up. WHO Classification: Normal. The patient’s hip was also scanned twice, also showing the variation of BMD values in the graph. **B **Example GE lunar left hip DXA. WHO Classification: Normal

The hip image should have an equal amount of soft tissue, approximately 3 centimeters, below the ischium and above the greater trochanter. To accomplish this, the ischium should first appear on the third sweep (pass of the machine arm) and should continue 2 full sweeps above the greater trochanter on GE instruments, and the cross-hairs from the Hologic position guide should be placed at the most lateral aspect of the greater trochanter. The lowest T-score at either the total hip or femoral neck may be used for diagnostic classification. The size and location of the hip ROI may vary depending on the specific protocol and manufacturer. Manufacturer-specific instructions must be followed for optimal positioning for all machines including Norland and Stratos. Care should be taken to replicate the ROI at all sites in the hip with subsequent scans to ensure precision over time.

##### Forearm (Fig. 4A and B)

 The distal cortex of the radius and the ulna should be visible. The edge detection should be checked. For both Hologic scan and GE scans, the top line of the global ROI is placed at the distal tip of the ulnar styloid process, which ensures correct placement of the one-third radius ROI. The ultra distal ROI may need to be moved to be below the radial endplate. Air must be visible on the ulnar side. In a GE scan, the index line is placed at the most distal tip of the ulnar styloid process, this determines where the software places the one-third radius ROI (analogous to one-third radius with Hologic). The ultra distal radius ROI may be adjusted to be just below the radial endplate. The one-third (33%) radius ROI is used for diagnosic classification. The demographics and database used in the analysis should be confirmed. On a follow-up forearm scan, the copy feature is used to make sure that the measurements are precise when compared to the prior scan.

**Fig. 4:**
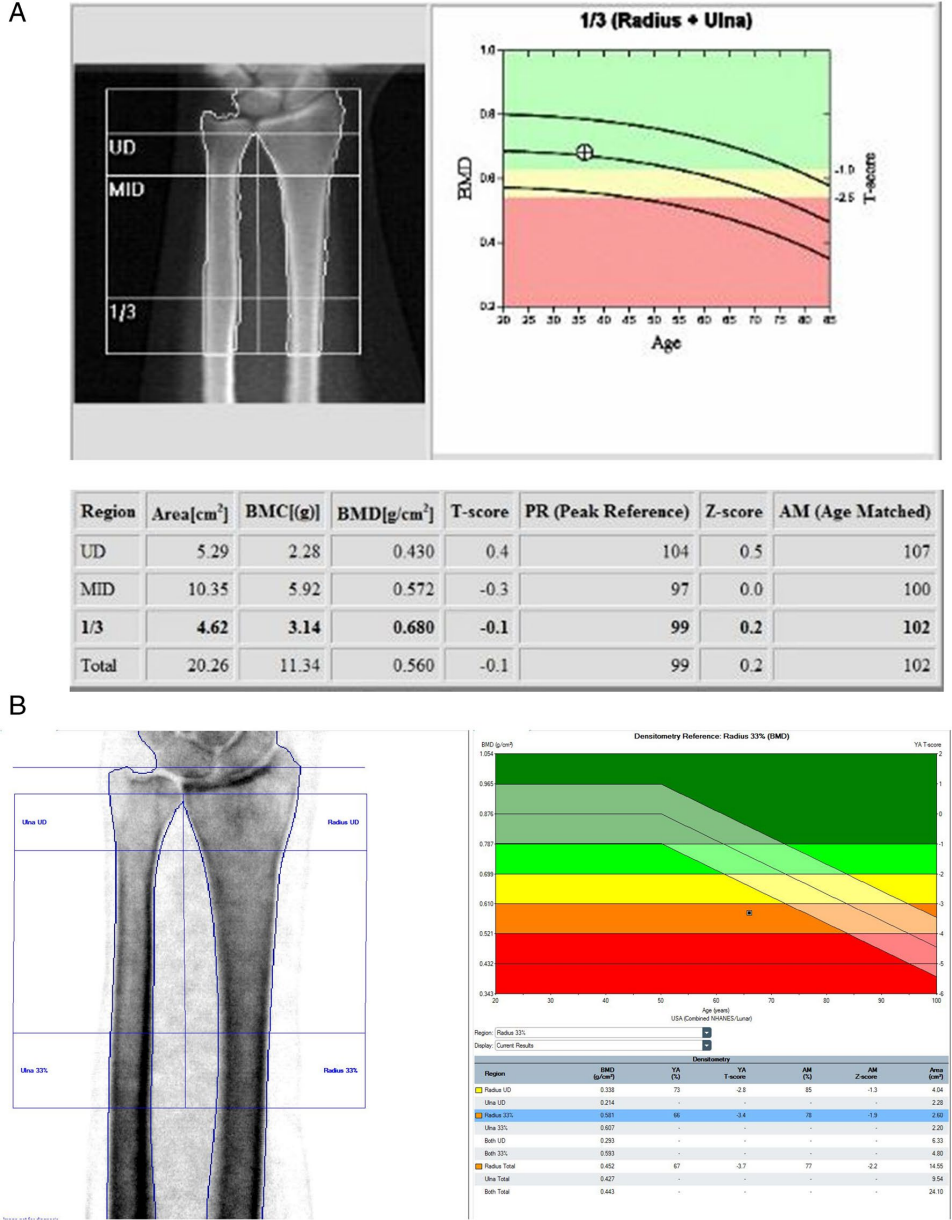
**A** Example Hologic Forearm 1/3 (33%) radius DXA. WHO classification: Normal. **B **Example GE Lunar Forearm—Radius- DXA. WHO Classification: Osteoporosis

The forearm analysis includes the total radius, one-third (33%) radius, and ultradistal radius. The one-third (33%) radius T-score in the nondominant forearm should be used for diagnostic classification and monitoring [4], but the latter should be taken with caution because it is a small bone area with poor measurement precision. Current guidelines do not recommend using ulnar BMD.

##### Vertebral fracture assessment (VFA) (Fig. 5)

 VFA utilises low radiation dose imaging of the lateral lumbar and thoracic spine that can be acquired at the time of BMD measurement on DXA. VFA is performed for the purpose of diagnosing vertebral fractures with the range of evaluable vertebral bodies from about T4 to L4. The scanning area should include the entire spine, starting from the S1 vertebral body upwards.

**Fig. 5:**
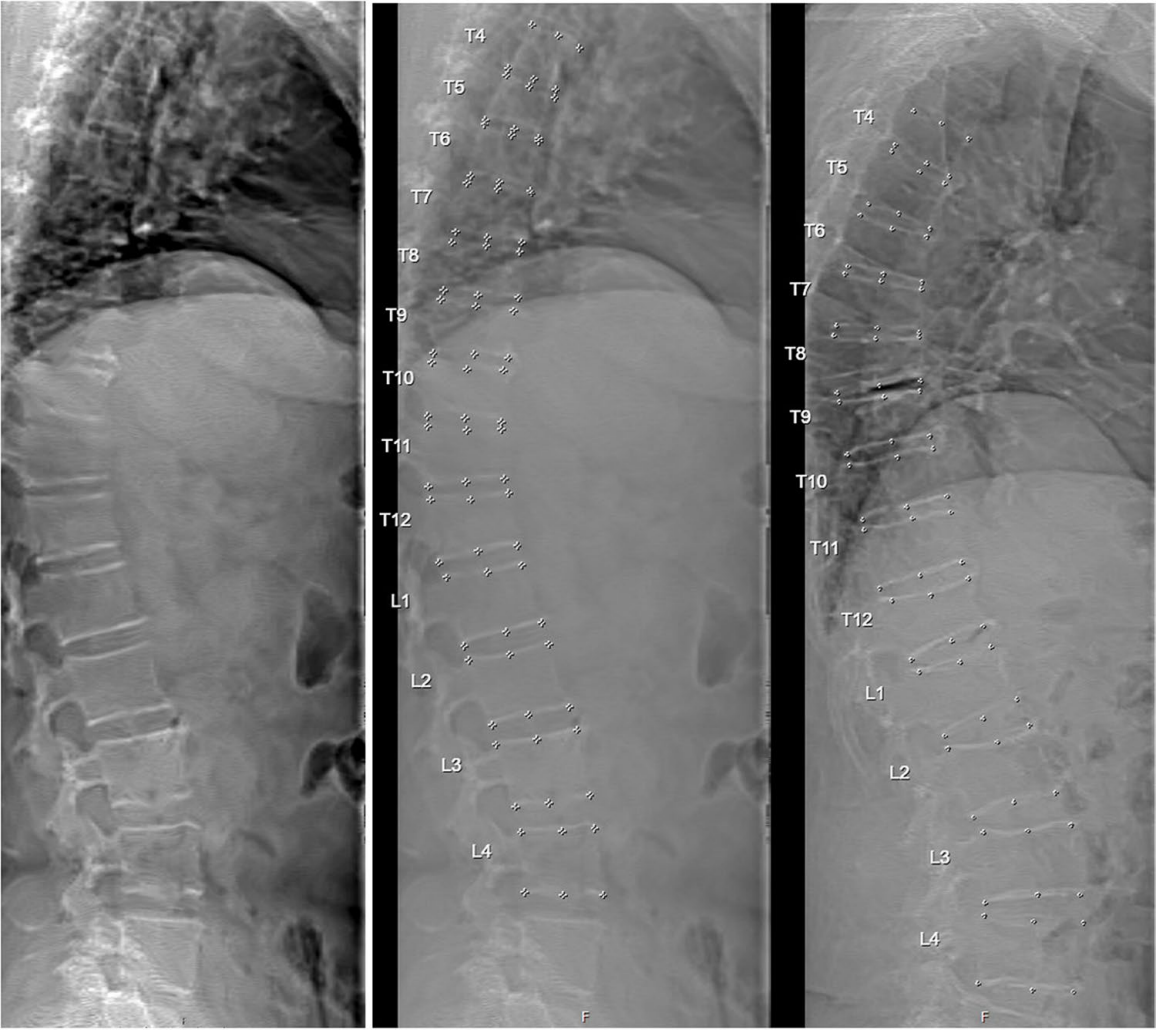
Examples Hologic VFA DXA. Mild fracture at the vertebral level Th11, grade 1 (left side and mid (same subject but with 6-point markers). Right-sided fractures with moderate wedge at level Th7 and Th8, and a moderate crush fracture at level Th12

Occasionally, it is difficult to determine the S1 level. In general, most individuals have 5 lumbar vertebrae with the iliac crests at the level of the L4/L5 disk, although in some individuals, there may be either 4 or 6 lumbar-like vertebral bodies. The interpreter must be familiar with standard scanning procedures in order to judge if the image and resulting data are acceptable. Depending on the scanner capabilities, scans may be acquired either in a lateral decubitus position or supine with the tube/detector in a lateral position (C-arm style scanners). Difficulties in positioning may arise in patients with mobility challenges, kyphosis or scoliosis, and may require repositioning (e.g. reverse the side of the lateral position to straighten the curve) or if justified on clinical grounds a traditional lumbar and thoracic lateral radiograph.

## Part II: Data interpretation, reporting DXA

### BMD interpretation

Absolute BMD values differ between the vendors due to inherent differences in how each technology works. However, for diagnostic classification, this value is converted into a T-score and/or Z-score value which is a patient’s BMD represented in standard deviations in comparison to a reference population, with a precision of 1 decimal place [4, 16]. The ISCD guidance recommends that T-scores are preferred in postmenopausal women, perimenopausal women, and men over the age age 50 years, whereas Z-scores for younger individuals. On the other hand the International Osteoporosis Foundations (IOF) supports the use of T-scores in premenopausal women and men under the age of 50 years [17] as well. As described in Part I, the standard skeletal sites for DXA measurement are the hip (total hip and femoral neck) with the femoral neck being designated by the IOF as the reference site for epidemiological studies [18], the L1-L4 region of the lumbar spine, and radius. However, in chronic arthritides such as rheumatoid arthritis, BMD should not be measured at the radius, because there is local, juxta-articular bone loss resulting in a lower BMD than in other parts of the skeleton. BMD in g/cm2 is used primarily for input into fracture risk algorithms (e.g., FRAX) and to monitor the skeletal effects of osteoporosis [4]. Incorporating ethnicity into FRAX (US, South Africa and Singapore versions) aims to help calibrate interventions appropriately, addressing racial disparities in fracture risk assessment and intervention thresholds. It is important to note that the significance of ethnicity varies by location; for instance, black individuals in the US exhibit lower fracture probabilities than Caucasians [19]. However, their fracture risk remains higher than that of African black individuals, partly due to differing fracture rates and lower mortality risks in the US population [20]. Despite the widespread acceptance of FRAX, it is advised that it be considered as an important reference platform rather than a definitive gold standard tool in fracture risk assessment [21]. Applying the World Health Organization (WHO) criteria, a T-score value less than or equal to -2.5 at the lumbar spine, one-third (33%) radius, femoral neck, or total hip is consistent with osteoporosis, while T-values ≥ -1.0 at these ROIs represent normal BMD [22, 23]. Diagnostic classification is based on the lowest T-score at any of the recommended DXA regions. Caution is advised when performing forearm measurements, as this is not the most relevant site for fracture risk assessment. A T-score between − 2.5 and − 1.0 is defined as “osteopenia”, “low bone mass” or “low bone density” Table 1) [4, 5, 23, 24]. In children and adolescents, premenopausal women, and men under the age of 50 years, the ISCD recommendation is to use Z-scores [23], although IOF has recommended use of T-scores in younger men and premenopausal women who are no longer growing [17]. In these younger populations, a Z-score ≤ -2.0 is defined by ISCD as “bone mineral density below the expected range for age,” with the suggestion that the terms osteopenia or osteoporosis should not be used to classify BMD measurements in these patients. In contrast, the IOF recommends that, in order to ensure consistency with the WHO operational definition of osteoporosis, a T-score ≤ -2.5 in premenopausal women and men younger than 50 years may be viewed as diagnostic of osteoporosis in the presence of skeletal fragility [17]. Indeed the IOF and ESCEO positions solely recognise this WHO operational definition as the clinical diagnostic criterion for osteoporosis, maintaining a distinction between diagnostic and intervention thresholds, and avoiding conflation of risk factor with outcome [25]. However, in recent years, some other societies (EANM, ASBMR and CSEM) have pragmatically proposed that a diagnosis of osteoporosis may be presumed in the presence of a prior low-trauma major osteoporotic fracture, even with a normal BMD (hip, spine, forearm, humerus, pelvis) [26]. This, however, does not exclude other sites, including fracture of the humerus, ribs, tibia (excluding the ankle) and other femoral fractures. It is also possible that the vertebral fracture sustained in the remote past may have been in association with a significant trauma. Historical information may be of value to clarify this.

**Table 1:** WHO definition of osteoporosis [27]

WHO classification	T-score
Normal	≥ -1.0
Low Bone Mass (Osteopenia)	< -1.0 to > -2.5
Osteoporosis	≤ -2.5
Severe osteoporosis	≤ -2.5 with fracture [2]

#### Reference database for BMD reporting

 The ISCD Official Positions [4, 5] recommend the use of uniform White (non-race adjusted) female reference database for the calculation of T-scores in women and men of all ethnic groups [23]. For the hip, many organizations (e.g., IOF, ESCEO, ISCD, WHO, BHOF) recommend the use of the NHANES III reference databases in White women aged 20–29 years [23, 24]. When this database is used, T-scores at the femoral neck and total hip are similar between manufacturers. Of note, the left hip is used in NHANES [12], on which the normative database for T-score is based. However for the lumbar spine and other skeletal sites, the database is manufacturer-specific. Clear examples of how they may differ are seen in the age-related curves on the normogram plots for the spine and forearm between GE and Hologic, shown in Figs. 2, 3, and 4. This may not be followed in all countries, and local application may vary, which will likely result in different T-scores and Z-scores. The T-scores and Z-scores are dependent on the measured BMD, the reference data utilized, the skeletal site being evaluated, as well as the method by which the T-score and Z-scores are derived, which can have a significant impact on the diagnosis [16, 28]. BMD values cannot be directly compared amongst different manufacturers. If local reference data are available, they may be used to calculate Z-scores [23].

The major DXA manufacturers have incorporated White based reference peak BMD to calculate T-scores. However, there is considerable variation in the attainment of peak BMD depending on the skeletal site, ethnicity and genetic and environmental influences [29]. It is also suggested that an Asian reference database is more appropriate for the Asian population [30, 31]. Thus, the use of different reference databases will influence the diagnosis of osteoporosis or low bone mass, leading to either underestimation or overestimation of low BMD [28]. For example if a white male normative reference database is used to calculate the T-score instead of a white female normative reference database will result in a higher prevalence of low bone mass and osteoporosis in men [12, 32]. Hence use of the young adult Caucasian female normative reference database or T-score calculation is recommended for both women and men.

#### Repeat BMD technical considerations

 When possible, repeat BMD measurements should be conducted in the same facility with the same DXA system, same software, same scan mode, and same patient positioning and same hip and forearm to enable precise and accurate comparisons over time. Additionally, scans should be obtained at a facility with a skilled DXA technologist who has performed precision assessment [4, 5, 33]. It is critical that both the initial and subsequent scans are of high quality and use the same protocol [34]. Modern DXA systems include a ‘copy’ feature and other modifications to assist those performing and analyzing the scan to ensure that repeat scans are comparable to measurement parameters used in the previous scan. Quantitative BMD comparison with serial measurments is based on absolute BMD values in g/cm^2^, and not T-scores or Z-scores [4, 5, 34]. Each centre should calculate its own measurement (precision) error for each skeletal site using 30 duplicate or 15 triplicate scans from a representative group of patients in their practice. This value, known as the least significant change (LSC), is calculated with a 95% level of confidence as 2.77 x the precision error [4, 5, 7]. The ISCD recommends evaluation of changes in BMD using absolute values, although reporting of changes is often expressed as percentages, which is easier for clinicians and patients to interpret [, 13,35. When the precision error is 1%, the LSC is 2.8% and when the precision error is 2% the LSC is 5.6%.

When using DXA to monitor BMD over time at the lumbar spine and total hip, only changes which meet or exceed the LSC should be considered significant and noted as such [4, 5, 7]. The ISCD recommends that the maximal acceptable LSC for a technologist is 5.0% for the total hip and 5.3% for the lumbar spine [4, 5, 7]. In clinical practice, the absolute change in BMD in gm/cm^2^ is preferred over % change for LSC [4]. Given variability in machines and systems from one imaging center to the next, the LSC from the manufacturer should not be used without confirmation. Similarly, a change in BMD between instruments that are not cross-calibrated cannot be reliably reported [4, 5] since it is not possible to know if BMD has actually changed, or if the change is solely due to measurement error in the absence of knowledge of the LSC. Finally, if a patient has a known diagnosis of osteoporosis based on prior imaging, a follow-up scan does not change the original diagnosis. Rather, the repeated imaging is used to monitor changes in BMD over time. Even if T-scores improve to > -2.5, the diagnosis of osteoporosis is durable. Preferable patients should return to the same DXA machine that was used to perform their most recent prior study, provided that the facility in vivo precision and LSC values are known and do not exceed established maximum values.

Alternatively, a DXA machine of one manufacturer enables comparison by using conversion factor for different machine [4, 5, 36]. See also part IV, Clinical Indications.

#### Vertebral fracture assessment (VFA)

 Dedicated software can be used to automatically place markers at the anterior, posterior and in the middle positions and the superior and inferior endplates of each vertebral body. Manual adjustment may be necessary when placement of digital 6-point markers is incorrect. If measurement of vertebral height is necessary, the fiducial points for an individual vertebral body can be initially placed by software with confirmation and correction of placement and vertebral levels should be labeled. The methodology utilized for vertebral fracture identification should be similar to standard radiological approaches and be provided in the report.

Knowledge of anatomy and variants regarding vertebral bodies is essential to adjust the automated interpretation of the VFA. Vertebral levels not adequately visualized should not be included in the analysis and should be noted as exclusions in the report. The morphology assessment performed must be validated by the operator/interpreter. When possible, reviewers should use pre-existing images to confirm whether a compression fracture is chronic or new. When there is doubt as to the presence of a vertebral fracture or an atypical-appearing vertebral body on VFA, additional imaging such as a lateral lumbar and thoracic radiograph should be performed to confirm the finding and/or exclude other pathologic processes.

The current clinical technique of choice for diagnosing and classifying the severity of VFs using VFA is the Genant visual semiquantitative (SQ) method [23, 37, 38]. Other methods have been proposed, such as the Algorithm-Based Qualitative (ABQ) Fracture Classification System, but are less commonly used [39]. Additional imaging following DXA-VFA with conventional radiographs and/or other imaging techniques may also be clinically indicated in individuals with suboptimal vertebral visualization, two or more mild (grade 1) without any moderate or severe (grade 2 or 3) deformities, equivocal fractures, lesions or vertebral deformities in patients with known or suspected malignancy, unidentifiable vertebrae between T4 and L4, sclerotic or lytic changes, or findings suggesting conditions other than osteoporosis [23].

Repeat VFA testing is indicated in patients at high risk for VFs following an initial VFA [23], in osteoporotic patients with new episodes of back pain, and in osteoporotic patients with height loss. A comparison of the diagnostic accuracy of DXA-VFA with that of spinal radiography for VFs showed a good agreement using the SQ approach [38]. Sensitivity and specificity vary between different models of DXA scanners [40], particularly in low-grade fractures (grade 1). The HD Instant Vertebral Fracture™ assessment dramatically improves the detection of VFs by doubling the resolution of previously available techniques with a low-dose, single-energy image [41]. Although adults with prevalent VFs are older and have lower BMD than those without, a U.S. NHANES study showed that some of those with fractures had normal spine and total hip T-scores, with a mean femoral neck T-score of -1.4. Among participants age ≥ 65 years with vertebral fractures, the proportion with osteoporosis by BMD criteria was 38% [41]. This suggests that eligibility criteria for VFA should sometimes include individuals with osteopenia or normal BMD.

#### Visualization of abdominal aortic calcifications (AAC)

**Fig. 6:**
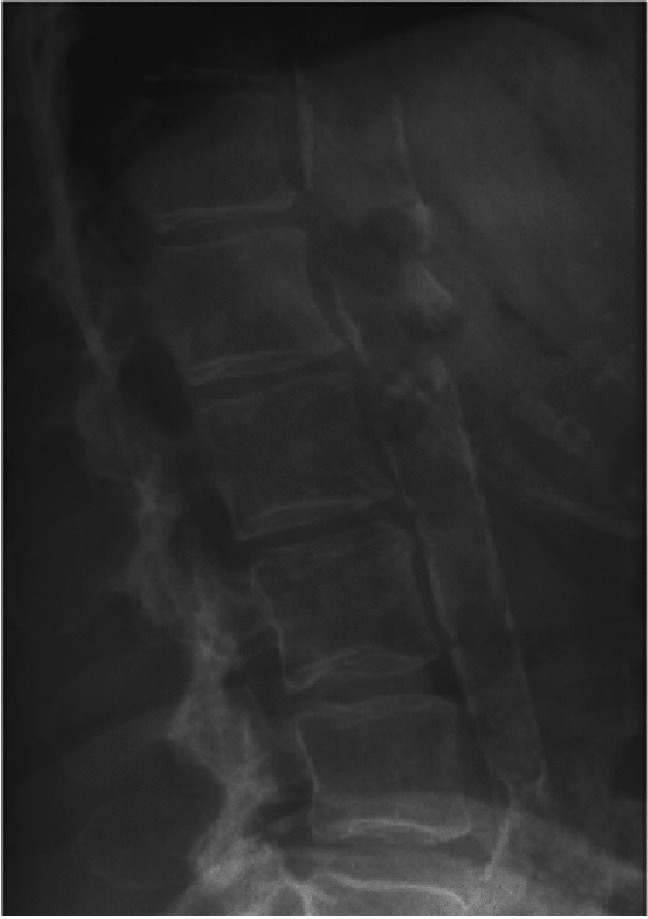
Abdominal aorta calcification (AAC) acquired with DXA

 In addition to the detection of VFs, DXA-VFA can be used for identification and scoring of abdominal aortic calcification (AAC) (Fig. 6). Evaluation of VFA images for AAC can be assessed (performed) manually using either 24-point AAC scale or simplified visual 8-point scale score system [42, 43]. A few recently published studies showed promising results with the use of machine learning techniques for automatically detecting and scoring AAC [44], with strong prediction of cardiovascular events [45–47].

#### Trabecular bone score (TBS)

**Fig. 7:**
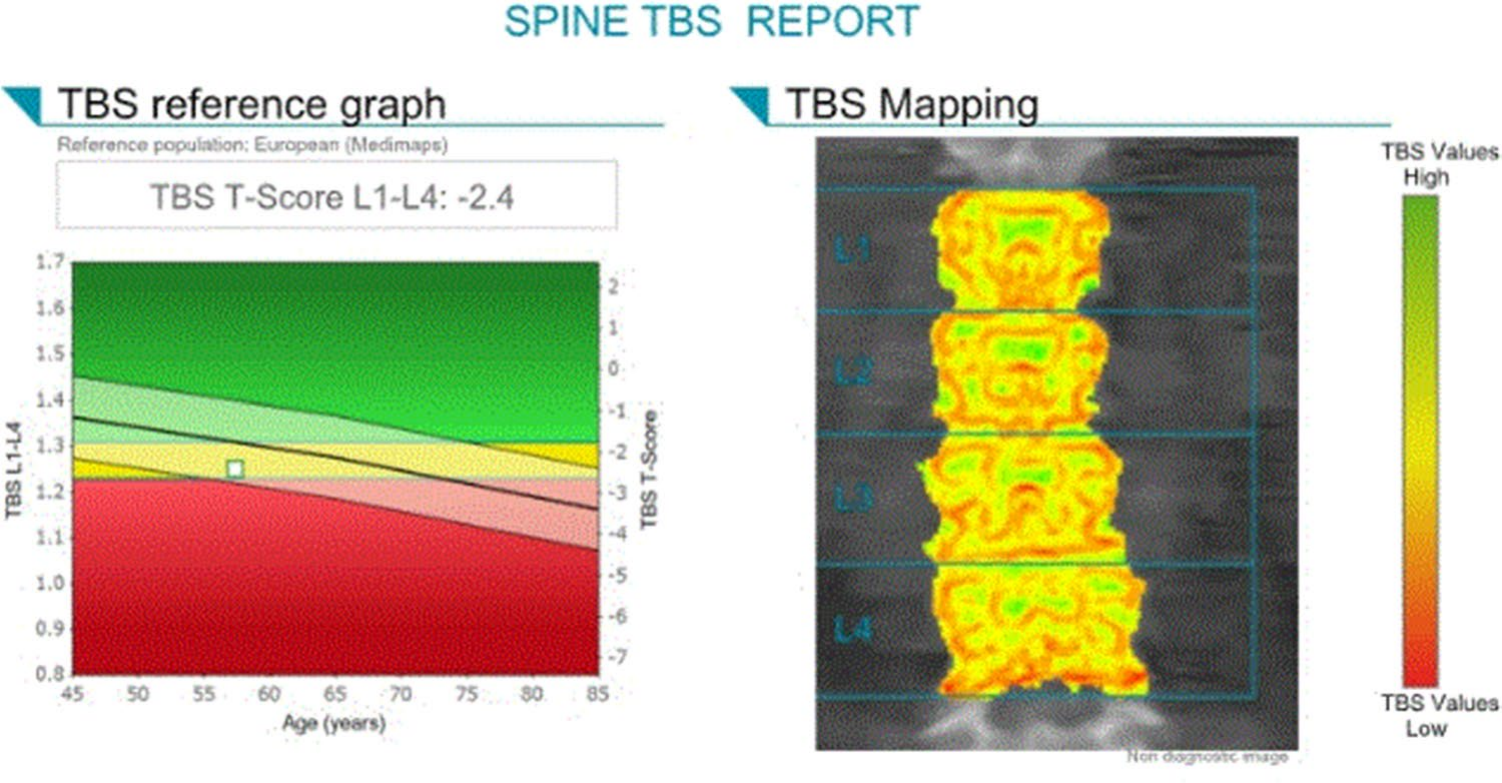
TBS example of a 61-year-old woman. Normative reference data in children and various ethnicities are limited

 The ISCD Official Positions [4] and a position paper from ESCEO [48] provide information on TBS use in clinical practice (Fig. 7). TBS) is a DXA –based software applied to lumbar spine DXA images previously obtained for BMD assessments [49, 50]. Another advantage is that TBS can be applied retrospectively to previously obtained DXA scans without the need for repeated testing [50]. TBS of the lumbar spine has been shown to predict the risk of fragility fractures independent of BMD and clinical risk factors in men and women over 50 years old. The greatest utility of TBS appears to be for those individuals who lie close to a FRAX or BMD T-score pharmacologic intervention threshold. As such, TBS should be used in conjunction with BMD and/or FRAX probability rather than as a standalone measure [23, 48]. TBS can be used to adjust either FRAX probability or BMD T-score, and is incorporated in theFRAXplus^®^ platform such that modification of FRAX probability to reflect a TBS measurement can be readily undertaken [5, 51].

TBS, used in combination with BMD, may add useful information in monitoring response to anabolic or long-term denosumab treatment, but is of limited value for bisphosphonate or short-term denosumab therapy [48, 52]. The clinical utility of monitoring with TBS is influenced by the LSC value. It is suggested that the DXA facility calculate a TBS LSC using the same methodology as that described for BMD LSC, or uses a conservative estimation for TBS LSC of 5.8%, based on the largest published value [52]. The presence of excess abdominal fat tissue may induce image noise that can artificially reduce TBS values. Therefore, it is recommended to perform TBS only in patients with a BMI of 15–37 kg/m^2^. To overcome the interference of abdominal soft tissue thickness (STT) on TBS a new TBS software algorithm that accounts for STT rather than BMI has been developed [53]. In individuals who have experienced significant weight change between DXA scans, change in TBS should be interpreted with caution.

#### Detection of incomplete atypical femur fractures (iAFFs) by DXA

 Incomplete atypical femur fractures are low trauma fractures characterized by focal periosteal or endosteal thickening of the lateral femoral cortex. Atypical fractures most commonly occur in the femur, and are rarely seen at other locations. The fractures are predominantly observed in bisphosphonate users, but may also occur in patients on denosumab, romosozumab and in non-users of osteoporosis pharmacotherapy [54].

**Fig. 8:**
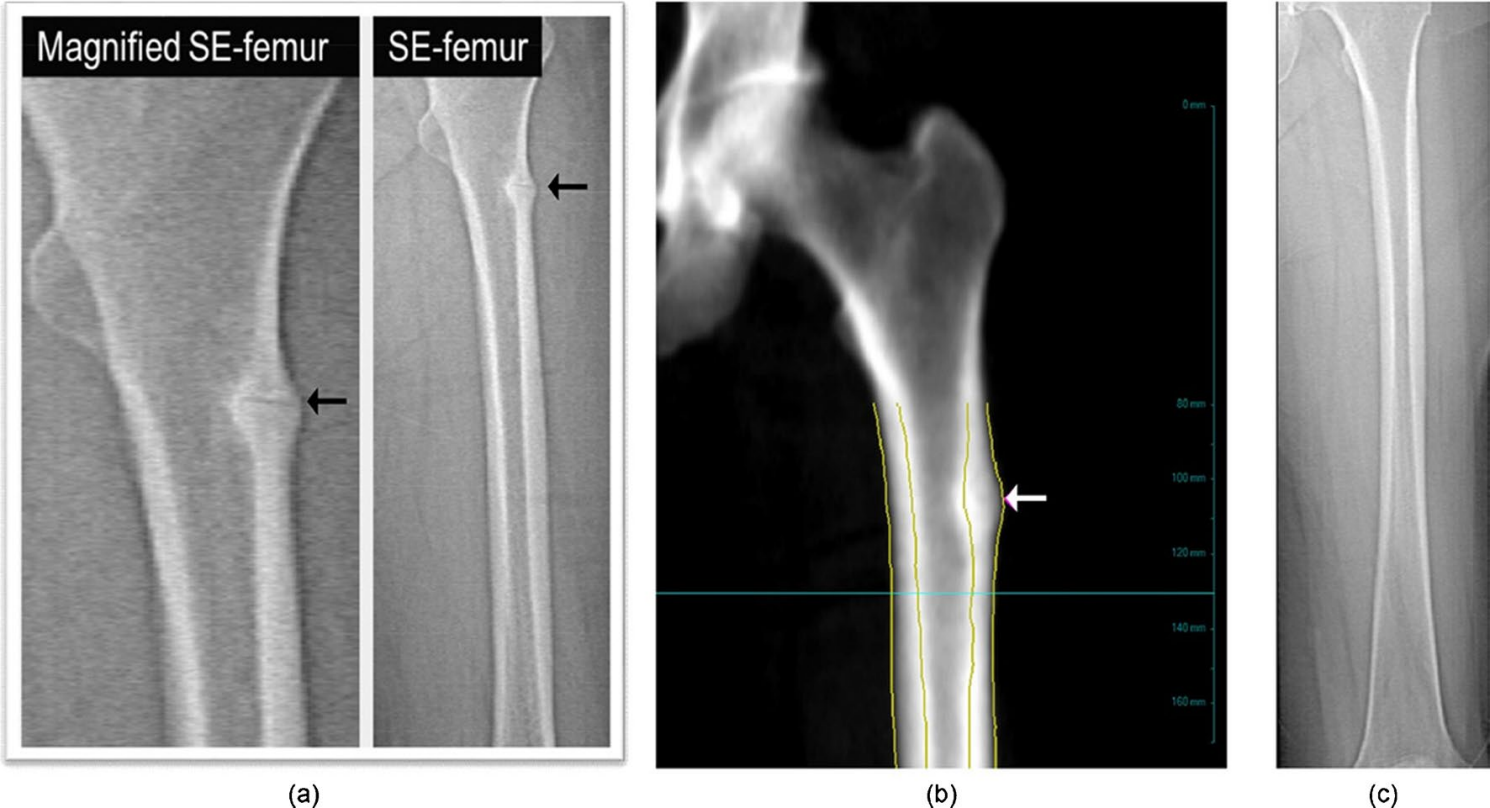
Densitometer-based femur imaging. **a** Single-energy scan showing beaking on extended-length femur imaging (arrows). **b** Dual-energy scan showing focal cortical periosteal and endosteal reactions at the lateral cortex (arrow). **c** Normal image from densitometer-based full-length femur imaging (FFI) with permission of Elsevier [55]

According to the ASBMR Task Force definition, iAFFs are stress fractures typically located below the lesser trochanter of the femur to the distal supracondylar flare (Fig. 8) [55]. iAFFs can be detected on DXA images or plain radiographs of the femur as an active lesion with a lucent line (“beaking”) in the middle of the cortical thickening [56].

Extended femur DXA scanning from the lesser trochanter to the supracondylar flare at the knee, or full-length femur imaging (FFI) methodology, is the preferred DXA mode for early identification of iAFFs [55, 57]. The majority of iAFFs involve the proximal femoral shaft, but they may occur at other locations including the distal femoral shaft and the entire femoral shaft should be visualized bilaterally, if possible.

The recommendations reported by the 2019 ISCD Official Positions paper on the detection of atypical femur fractures are as follows:

1. Femur DXA images should be reviewed for iAFFs using either default-length femur imaging or FFI.
2. For the detection of abnormalities in the spectrum of iAFF, bilateral default-length femur imaging or FFI images should be used; the presence of cortical thickening, with or without a lucent line, should be reported. In suspected iAFFs, further imaging using X-ray, or sometimes computed tomography (CT), magnetic resonance imaging (MRI), or an isotope bone scan is needed to determine the etiology of the lesion.

Currently, iAFF detection is not commonly used in clinical practice, although newer scanners more commonly offer this helpful software component for patient assessment [5, 55, 58, 59].

#### Whole body composition

**Fig. 9:**
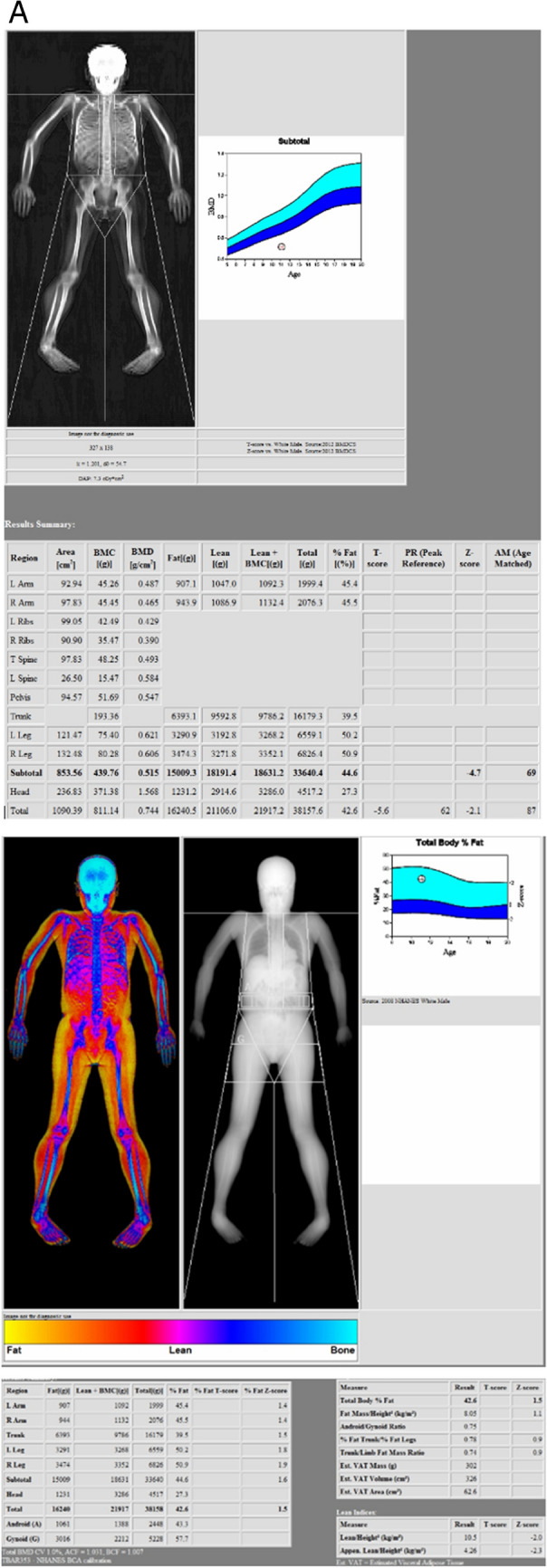

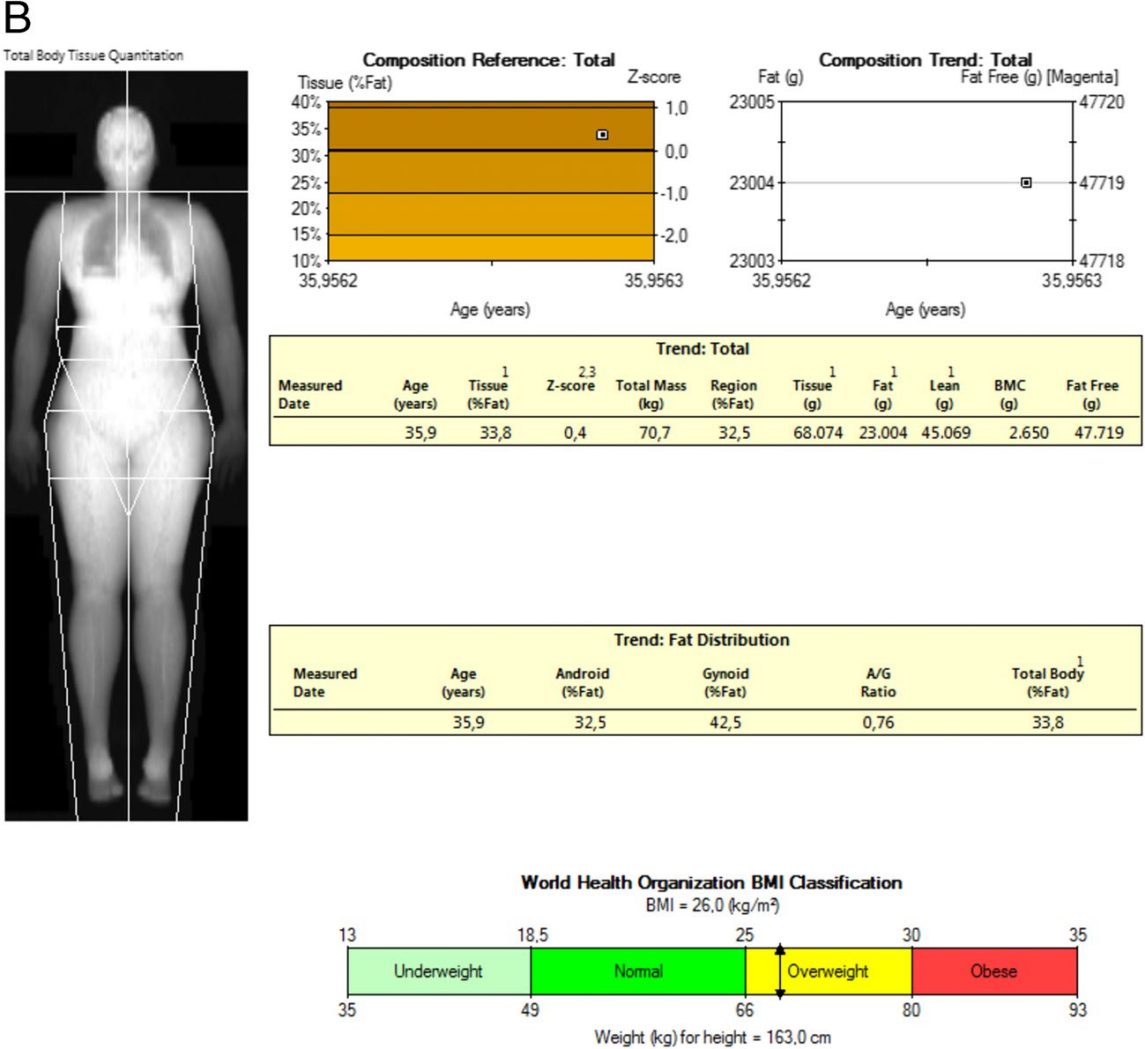
**A **Top and bottom: example WBC Hologic of a 11-year-old boy with Duchenne muscular dystrophy and glucocorticoid therapy. **B **Example WBC GE LUNAR of an adult

 The assessment of whole-body composition (WBC) by DXA enables measurement of total and regional BMC and BMD and lean and fat mass (Fig. 9A and B). In order to compare results across manufacturers, in vivo cross-calibration is necessary [4]. For cross-calibrating systems of the same manufacturer and model, an appropriate whole body phantom may be used. One technologist can do 10 whole body phantom scans with repositioning. If a difference in mean fat mass or lean mass percentage greater than 2% is observed, the manufacturer should be contacted for service/correction.

The WBC scanning procedure should be performed as follows: positioning of the arms, hands, legs and feet whenever possible should be performed according to the NHANES method (palms down, hands isolated from the body, feet neutral, ankles strapped, arms straight or slightly angled, face up with neutral chin) [60]. The manufacturer’s recommendations for ROI placement and artifact removal should be used. For adults, total body (with head) values of BMI, BMD, BMC, total mass, total lean mass, total fat mass, and percent fat mass should appear on all reports [4] Total body BMC as represented in the NHANES 1999–2004 reference data should be used when using DXA in 4-compartment models, and is appropriate for different races, both sexes, and for ages from 8 to 85 years. DXA measures of adiposity and lean mass include visceral adipose tissue (VAT), appendicular lean mass index (ALMI: appendicular lean mass/height^2^), android/gynoid percent fat mass ratio, trunk to leg fat mass ratio, lean mass index (LMI: total lean mass/height^2^), fat mass index (FMI: fat mass/height^2^). Both Z-scores and percentiles are appropriate report outputs if derived using methods to adjust for non-normality. The use of DXA adiposity measures (percent fat mass or fat mass index) may be useful in risk-stratifying patients for cardio-metabolic outcomes. Specific thresholds to define obesity have not been established. “Low lean mass” could be defined using appendicular lean mass divided by height squared (ALM/height^2^) with Z-scores derived from a young adult, race, and sex-matched population. Currently, WBC is not a routine clinical application, but can useful in selected population. Thresholds for low lean mass from consensus guidelines for sarcopenia await confirmation [5].

#### Components of the final DXA report

 The final DXA and VFA reports should follow a standard template and may include recommendations on additional diagnostic work-up (e.g. radiograph of the spine, evaluation of secondary causes of osteoporosis) and indications for treatment. See Tables 2 and 3 and the ISCD website (https://www.iscd.org/certify/accreditation/dxa-report-examples).

**Table 2:** Components of DXA report [4, 5, 61https://www.iscd.org/certify/accreditation/dxa-report-examples]

Standard DXA report components	Example of DXA report
	*Previous DXA scan: None*
Name	*J.K.*
Medical record ID number	*XX321*
Date of birth/age	*66 years*
Sex	*Female*
Body weight Body height	*70 kg 168 cm*
Menopausal status/age at menopause	*Postmenopausal /age at menopause 52 years*
Requesting provider	*Gynaecologist*
Indications for the test	*Postmenopausal woman age 65 years or older*
Manufacturer and model of instrument and software used	*Hologic Delphi C*
• Technical quality and limitations of the study, stating why a specific site of ROI is invalid or not included. • BMD in g/cm^2^ for each site. • The skeletal sites, ROI, and, if appropriate, the side, that were scanned. • The T-score and/or Z-score where appropriate. • WHO criteria for diagnosis in postmenopausal women and in men age 50 years and over. • Interval change (if a follow-up study)	***BMD results***: *good quality* ***Proximal femur***: *Total hip BMD/T-score*: *0.809 g/cm*^*2*^*/ -1.1* *Femoral neck BMD/T-score*: *0.680 g/cm*^*2*^*/ -1.5* *Comments: Due to a previous left hip replacement*,* scanning of the right hip was performed* ***Lumbar spine***: *Total L1-L4 BMD/T-score*: *0.958 g/cm*^*2*^*/ -0.8* *Comments: there are mild degenerative changes*
• Risk factors including information regarding previous non-traumatic fractures. • A statement about fracture risk [62]. Any use of relative fracture risk must specify the population of comparison (e.g., young-adult or age-matched, race/ethnicity) [63]. Reporting of absolute fracture risk is preferred. Identify the fracture risk calculator used. Include positive fracture risk components that were included in the calculation. • A general statement that a medical evaluation for secondary causes of low BMD may be appropriate. • Reports should contain a statement describing why acquired exams were not reported or when a technically acceptable DXA exam has aspects that might confound BMD results. • Diagnostic classification is an essential component of the report, with application of the WHO diagnostic criteria when appropriate. • When reporting or referring to race, “White” is preferred to “Caucasian”. • Recommendations for the necessity and timing of the next BMD study • Recommendations for further non-BMD imaging	*Risk factors*: *current smoking* ***Fracture risk***: *FRAX 1.5% risk of hip fracture and 15% risk of major osteoporotic fracture* ***CONCLUSIONS***: *Diagnosis: Osteopenia (low bone mass)* *Evaluation of secondary causes of low BMD: suggested* *Treatment recommendations: based on clinical circumstances and national clinical guidelines* *Follow-up DXA: 2–3 years or sooner if clinically indicated* *Other recommendations*:

**Table 3:** Components of VFA report

Standard VFA report components	Example of VFA report
	*Previous VFA: None*
Name, age	
Referring physician	
Indication(s) for the study	
Technical quality	*Good*
**VFA report interpretation**: • Unevaluable vertebrae • Deformed vertebrae, and whether or not the deformities are consistent with vertebral fracture using a standardized methodology • Unexplained vertebral and extravertebral pathology, e.g. AAC • Optional components include: fracture risk, AAC and recommendations for additional studies.	*Th4-L4 vertebrae evaluated.* *Moderate wedge fracture (25–40%) at level thoracic 7.* *One mild biconcave fracture at level lumbar 1.* *Calcification of the abdominal aorta.* ***Conclusion***: *2 vertebral fractures. For confirmation a conventional spinal radiograph can be considered.*
**Follow-up VFA report**: • Comparability of studies and clinical significance of changes, if any.	

Overall, inadequate awareness and knowledge of potential pitfalls and nonadherence to the ISCD Official Positions in DXA interpretation may lead to inaccuracies in BMD readings [35]. Aiming to reduce reporting errors, a DXA interpretation template based on ISCD recommendations has been developed [https://www.iscd.org/certify/accreditation/dxa-report-examples; 61]. Implementing a DXA reporting template may reduce major errors, shorten reporting time and improve report quality [61, 64].

DXA Reporting [5]:

- The acquisition of non-dominant/left hip or is appropriate to generate data for reporting T-scores (or Z-scores).
- When both hips have been scanned, the lowest T-score (or Z-score) of the right or left femoral neck or total hip should be used for diagnostic classification, but not the mean T-score (or Z-score).
- When both hips have been scanned on repeat tests, mean bilateral total hip BMD should be used for monitoring.
- Preferred terminology is to use “hip” when describing the site instead of ” femur” or “total proximal femur”. Use ”bilateral hips” when referring to both hips.

DXA Reporting: Reporting Fewer Than Four Vertebrae

- We do not recommend using a single vertebral body for diagnostic classification or for monitoring.
- Precision worsens progressively with fewer than 4 vertebral bodies included, whether contiguous or non-contiguous. The LSC should be modified according to the precision assessment for corresponding combinations of fewer than 4 vertebrae.

DXA Reporting: Reporting Results from Full-Femur Imaging (FFI)

- FFI is considered a screening tool for iAFFs.
- Clinical assessment of prodromal symptoms (pain) is not required for assessment of FFI.
- Focal lateral cortical thickening and transverse lucencies should be reported when identified on FFI.
- When both focal lateral cortical thickening and a transverse lucent line are present, there is a high likelihood for an iAFF.
- Diffuse cortical thickening alone is non-specific for an iAFF.
- Suggestions for Reporting of FFI (based on features):
  - NON-DIAGNOSTIC: Images are inadequate either due to acquisition issues, artifact or other patient factors. Consider dedicated radiographs to evaluate patient if necessary.
  - LOW likelihood features: Isolated diffuse cortical thickening, or no findings. Clinical correlation to decide if dedicated radiographs are necessary.
  - MODERATE likelihood features: Questionable focal lateral cortical thickening without a transverse lucent line. Clinical correlation and dedicated radiographs for clarification.
  - HIGH likelihood features: Definite focal lateral cortical thickening and a transverse lucent line. Urgent consultation and further imaging are recommended.

DXA Reporting: Quality Assurance

- Implement an internal program of peer-learning, following accepted radiologic practice, to facilitate quality reporting.

## Part III: pitfalls DXA/VFA

Pitfalls in DXA and VFA data generation are common, with errors due to patient positioning, data analysis, artifacts, and/or demographics. When DXA measurements are performed or reported incorrectly, there can be serious implications for osteoporosis diagnosis, management and monitoring with serial assessments. Physicians involved in performing and interpretating DXA and VFA should be familiar with the potential pitfalls to minimize errors and allow proper use of bone densitometry and VFA.

### Common pitfalls and artifacts in lumbar spine DXA [7, 65]

 Optimal interpretation of a DXA scan involves evaluation of the images and ancillary data. As in interpretating a chest radiograph or an electrocardiogram, it is useful to develop a “flow” for DXA interpretation. Items to be included in the interpretation contained in the following acronym: **PARED** (positioning, artifacts, regions of interest, edge detection, demographics), originally developed during the ISCD US LOC Quality Bone Densitometry course. To date the acronym, PARED has been utilized to guide interpretation of DXA studies [4, 5]. The IWG recommends the following modification of this strategy to include demographics.

**P** – **P**ositioning – Is the positioning of the patient correct?

**A** - **A**rtifacts – Are there any artifacts present within the region of interest scanned or in the soft tissue?

**R** - **R**egions of Interest – Are the regions of interest correct? On a follow-up scan are the regions of interest analogous?

**E** - **E**dge Detection – Is the edge detection correct?

**D** – **D**emographics (patient and risk factors for fracture) [66] and **D**atabase. Are the demographics properly recorded, including risk factors for fracture and is the correct database for comparison used?

The lumbar spine is in general the site most frequently affected by artifacts that may bias BMD estimates. In most cases, artifacts in the lumbar spine will cause a spurious increase in BMD values (as occurs in osteoarthritis), while a spurious decrease in BMD is less common [67].

### Spine osteoarthritis

 In (facet) osteoarthritis, osteophytes, hypertrophy and sclerosis of the facet joints develop and may cause increase in BMD. Approximately 40% of women aged 55 and 85% of those age > 75 years will have spine osteoarthritis. Consequently, vertebrae affected by significant structural changes or differing by more than a T-score of 1.0 from the adjacent vertebra should be excluded.

### Vertebral fractures

 Vertebral fractures typically occur at the thoraco-lumbar transition (T12-L2 region). A fractured vertebra demonstrates increased BMD values due to trabecular impaction and condensation associated with the fracture, with a mean BMD increase of 0.070 g/cm^2^ reported [68]. A fractured vertebra can usually be recognized on DXA by the reduced height, low area values, and linearly increased sclerosis. Nevertheless, the presence of vertebral fractures is not always easy to detect on DXA images, especially in the case of low-grade fractures. Therefore, in cases of uncertainty, it is recommended to check previous plain spine radiographs or other imaging (CT, MRI), if available, or obtain de novo radiographic spine images to verify the findings on DXA.

### *Other lumbar spine artifacts*:

- Vertebral augmentation cement.
- Surgical material and hardware projecting on the vertebral body (e.g., metal clips or wires, spine fusion material, scoliosis surgery rods and hooks).
- Implantable devices (e.g., pain pumps, neurostimulators).
- Ankylosing spondylitis.
- Radio-opaque contrast material for example from recent GI or GU studies.
- Undisolved calcium tablets.
- Bone metastases.
- Diffuse idiopathic skeletal hyperostosis (DISH).
- Splenomegaly due to glycogen storage disease.
- Incorrect counting of the lumbar vertebrae.
- A T-score difference between vertebrae ≥ 1.0.
- Aortic calcifications.

### Common pitfalls and artifacts in hip DXA

Patients are advised to have the test on their clothes, removing only metal parts, but thick or reflecting materials, could alter results [69, 70].

### Hip osteoarthritis

 In advanced cases of osteoarthritis, cortical bone thickening on the medial or lateral side of the femoral neck can occur, resulting in increased BMD values at the neck, but in general much less common as compared to the lumbar spine.

### Arthroplasty or osteosynthesis hardware

 The presence of hip prostheses or screws makes the site unsuitable for diagnostic purposes, although software can be used to assess periprosthetic BMD around the metal stem [71]. Therefore, the contralateral side should be used.

### Other hip artifacts

- Prior history of osteosynthesis hardware. Even after removal, sclerotic changes can remain visible in the femoral neck.
- Gluteal implants overlapping with bone structures. In the majority of these cases, the hip is not suitable for BMD assessment.
- Hip dysplasia.
- Bone metastases.
- Paget’s disease.
- Incorrect ROI position of the hip region.
- Incorrect rotation of the femur (lesser trochanter not visible).

A limitation of DXA scans is that they are based on two-dimensional projection images that measure BMD as the mass of bone per unit area, as this may underestimate true volumetric bone density in short or overestimate in tall adults. For this reason, they do not separate the effects of true bone density (i.e. grams of bone per unit volume) from those of bone size [72].

## Part IV: Clinical indications for DXA and VFA

The majority of international guidelines incorporate a case finding strategy based on clinical risk factors. Although screening by DXA is nominally recommended in the USA for all women aged 65 years or older, and men aged 70 years or older, in practice there is little systematic application of this policy. The MRC SCOOP study in the UK demonstrated a 28% reduction in the risk of hip fracture in a large multicentre randomised controlled trial of primary care screening, using FRAX hip fracture probability [73]. Meta-analysis of these findings with trials in the Netherlands and Denmark have demonstrated evidence for screening efficacy [74] and informed a recent IOF position paper supporting implementation of screening for high fracture risk [75]. Various approaches have been suggested internationally to optimise screening efficiency; strategies such as targeting older individuals and use of automated systems which identify those at high risk from primary care records, offer potential ways forward [75–77].

For younger adults, DXA testing is recommended for those with a previous fracture or other major risk factors for osteoporosis and fracture [78] such as chronic glucocorticoid use or hypogonadism, (see Tables 4 and 5, and the Statements based on IWG consensus). Other risk factors include lifestyle choices, medical conditions and medications associated with increased fracture risk or accelerated bone loss or those taking or being considered for osteoporosis treatment [4, 35, 79]. A comprehensive list has been developed by the IWG [2].

**Table 4:** Indications for bone mineral density measurements multisociety

All women 65 years of age or older (IOF: guided by FRAX probability [80]
Postmenopausal women < 65 years with additional risk factors (ISCD [4], ACR [79] USPSTF [81], ACOG [82], NICE [83] or guided by FRAX probability (IOF [80]) ≥ 50 years AACE [35] ≥ 50 years with degenerative changes (ACR) ≥ 50 years with non-traumatic fractures (ACR)
Postmenopausal women with osteopenia identified radiographically (AACE)
Women during a menopausal transition who have one or more risk factors for osteoporosis (ISCD)
Premenopausal women or men < 50 years with risk factors (ACR)
Men ≥ 70 years (ISCD, BHOF [84], ACR) Men ≥ 75 years (NICE)
Men < 70 years with risk factors (ISCD, BHOF, ACR); Men < 75 years with risk factors (NICE)
Postmenopausal women and men aged 50–69 years based on risk profile (BHOF) Postmenopausal women and men aged ≥ 50 years with history of adult-age fracture (BHOF)
Individuals of any sex or age who develop one or more insufficiency fractures (ACR)
High FRAX score calculated without BMD (NOGG [85])
Osteoporosis risk assessment tools- OST, SCORE, OSIRIS, ORAI, body weight criterium [86].

*IOF* International Osteoporosis Foundation, *FRAX* Fracture Risk Assessment Tool, *ISCD* International Society for Clinical Densitometry, *ACR* American College of Radiology, *USPSTF* US Preventive Services Task Force, *ACOG* American College of Obstetricians and Gynecologists, *NICE* The National Institute for Health and Care Excellence, *AACE* American Association of Clinical Endocrinologists, *BHOF* Bone Health & Osteoporosis Foundation, *NOGG* National Osteoporosis Guideline Group, *OST* Osteoporosis Self-Assessment Tool for Women, *OSIRIS* Osteoporosis Index of Risk, *SCORE* Simple Calculated Osteoporosis Risk Estimation, *ORAI* The Osteoporosis Risk Assessment Instrument

**Table 5:** Indications for vertebral fracture assessment or standard radiography

ISCD [5, 23]
T-score < -1.0 with one or more of the following: • Women aged ≥ 70 years or men aged ≥ 80 years • Historical height loss > 4 cm (>1.5 inches) • Self-reported but undocumented prior vertebral fracture • Oral glucocorticoid therapy equivalent to ≥ 5 mg of prednisone or equivalent per day for ≥ 3 months
BHOF [84]
• Women aged 65 years and older if T-score is less than or equal to – 1.0 at the femoral neck • Women aged 70 years or older and men aged 80 years or older if T-score is less than or equal to – 1.0 at the lumbar spine, total hip, or femoral neck • Men aged 70–79 years if T-score is less than or equal to – 1.5 at the lumbar spine, total hip, or femoral neck • Postmenopausal women and men aged ≥ 50 years with the following specific risk factors [84]: • Fracture(s) during adulthood (any cause) • Historical height loss of ≥ 1.5 in. (defined as the difference between the current height and peak height) [87] • Prospective height loss of ≥ 0.8 inch. (defined as the difference between the current height and last documented height measurement). • Recent or ongoing long-term glucocorticoid treatment. • Medical conditions associated with bone loss such as hyperparathyroidism
NOGG
• Postmenopausal women, and men age ≥50 years, if there is a history of ≥4cm height loss, kyphosis, recent or current long-term oral glucocorticoid therapy • BMD T-score ≤-2.5 at either the spine or hip • in cases of acute onset back pain with risk factors for osteoporosis

*ISCD* International Society for Clinical Densitometry, *BHOF* Bone Health & Osteoporosis Foundation, *NOGG* National Osteoporosis Guideline Group

### DXA in Cancer patients

Cancer patients receiving specific antineoplastic treatments, particularly with the use of endocrine therapy, are at an increased risk of accelerated bone loss

#### Breast cancer

The updated guidance on the management of cancer treatment-induced bone loss in breast cancer (BC) patients recommends initial bone density measurement using DXA. In all postmenopausal and osteopenic premenopausal BC patients initiating aromatase inhibitors (AIs), VFA along with DXA could be a part of baseline or follow-up bone health evaluation. Joint use of TBS and BMD suggests a better prediction of fracture risk; however, the evidence in this regard is lacking in BC patients [88, 89]

Follow-up DXA measurement should be performed one year after initiation of AIs or every 2 years after starting anti-resorptive therapy. In case of an annual BMD loss of > 5%, BMD re-assessment regarding anti-resorptive therapy is required [89].

#### Prostate cancer

Androgen deprivation therapy (ADT) is associated with a 3–5% annual decline in BMD in men with prostate cancer, and increased fracture risk [90, 91]. A screening and clinical assessment at 1- to 2-year intervals during follow-up is required for risk stratification and estimation of the need for pharmacologic treatment [92]. The use of FRAX in men with prostate cancer does not include a specific correction for use of ADT, therefore “secondary osteoporosis” can be used for fracture risk estimation when femoral neck BMD is not available [93].

#### DXA in secondary osteoporosis

Exclusion of secondary causes of osteoporosis is necessary since the treatment of bone disease in these patients may require management of both the underlying condition and the skeleton itself [94]. However, the majority of current clinical practice guidelines for osteoporosis neglect secondary causes and focus on postmenopausal osteoporosis [92]. In patients with osteoporosis, a secondary cause is found in up to 30% of postmenopausal women, > 50% of premenopausal women, and between 50% and 80% of men, depending on the practice setting and diagnostic cutpoints [95].

Bone density assessment using DXA may underestimate fracture risk in some chronic diseases such as glucocorticoid-induced osteoporosis and type 2 diabetes. Notably, the variable secondary osteoporosis only affects FRAX estimations when BMD is not entered, but not when BMD is included, though rheumatoid arthritis and glucocorticoids are not subject to this [96].

#### Spinal Cord Injury (SCI) 4]

All adults with spinal cord injuries resulting in permanent motor or sensory dysfunction should have a DXA scan of the total hip, proximal tibia and distal femur (which can only be performed on certain DXA machines), as soon as medically stable. Serial DXA assessment of treatment effectiveness among individuals with SCI should include evaluation at the total hip, distal femur, and proximal tibia, following a minimum of 12 months of therapy at 1- to 2-year intervals [4]. Referral to a specialized centre should be considered.

#### Transgender persons [97–99]

Screening for osteoporosis should be based on the assessment of clinical factors including hormone therapy compliance, gonadal removal, and additional osteoporosis risk factors. Post-pubertal trans children and adolescents on gonadotropin-releasing hormone without sex steroid hormone therapy may be at risk for decreasing bone density. The Z-score in transgender individuals should be calculated using the reference data (mean and standard deviation) of the gender conforming with the individual’s gender identity. In gender nonconforming individuals, the reference data for the sex recorded at birth should be used. If the referring provider or the individual requests, a set of “male” and “female” Z-scores can be provided, calculating the Z-score against male and female reference data, respectively. Referral to a specialized centre should be considered. As discussed earlier, using a gender-specific reference for Z-score calculation can have significant effect on the diagnostic classification [28].

#### Repeat BMD and VFA measurements, clinical indications [4, 5]

Repeat BMD testing in combination with clinical assessment of fracture risk, bone turnover markers, if available, and other factors, including height loss and trabecular bone score, can be used to determine whether treatment should be initiated in untreated individuals. The BMD repeat testing interval depends on the overall clinical need as well as the baseline value [34, 100, 101]. For example, a patient who is taking high dose glucocorticoids may lose bone much more rapidly, particularly in the setting of cancer and active rheumatoid arthritis. Those with low BMD and rapid loss are among those at greatest risk of fracture [34, 101]. On the other hand, less frequent assessment may be appropriate in older individulas [34, 101]. A US study of almost 5,000 postmenopausal women aged ≥ 67 years demonstrated that the average time to transition to a densitometric diagnosis of osteoporosis (T-score ≤ -2.5) was 17 years for those with a T-score > -1.5, 5 years for women with T-score between − 1.50 and − 1.99, and 1 year for women with a T-score between − 2.0 and − 2.49) for 10% of the total population considered [100]. These data provided some guidance on appropriate intervals for BMD testing in untreated individuals.

Follow-up VFA or radiographic lateral spine imaging should be used in patients with continued high risk [e.g., historical height loss > 4 cm (> 1.5 inches)], self-reported but undocumented vertebral fracture, or glucocorticoid therapy equivalent to ≥ 5 mg of prednisone or equivalent per day for greater than or equal to three months) [5].

When used to monitor the effects of treatment, the frequency of BMD measurements should be guided by a number of factors, including the therapy used, availability of resources, national guidelines and reimbursement policies, and the clinical site being measured. Changes are generally greater at the lumbar spine than total hip and greater for osteoanabolic therapy than antiresorptive therapy. These data suggest the most likely result will be ‘no change’ for non-anabolic treatments if a DXA scan is repeated within 2–3 years from the beginning of anti-resorptive therapy [34, 101]. In addition, an incident fragility fracture while on treatment is generally regarded as an indication for bone densitometry. Based on the ISCD minimum acceptable LSC of 5% for the total hip and 5.3% for the lumbar spine, significant changes in BMD are unlikely to be seen before 3 years in individuals treated with most anti-resorptive drugs but may be evident in the LS after one year with anabolic therapies [101].

The latest ISCD 2023 update adds the following [5]:

- Follow-up BMD testing can aid in monitoring response to therapy.
- Follow-up BMD testing should be undertaken with clearly defined objectives and when the results are likely to influence patient management.
- Follow-up BMD testing should be performed if a fracture has occurred or new risk factors have developed, but should not delay treatment for secondary fracture prevention.
- Repeat BMD testing should be used to monitor individuals prior to a temporary cessation of bisphosphonate therapy and during the period of planned interruption of treatment.
- Repeat BMD testing intervals must be individualized considering an individual’s age, baseline BMD, the type of pharmacological treatment, and the presence of clinical factors which are associated with bone loss.
- Shorter intervals between BMD testing may be indicated in the presence of factors associated with rapid change in bone mineral density. Examples include the use of certain medications such as glucocorticoids, aromatase inhibitors, androgen deprivation therapy, and osteoanabolic therapies, medical disorders such as malabsorption and severe systemic inflammatory diseases, and other conditions such as prolonged immobilization, bariatric surgery, and surgical menopause.
- If changes in BMD are outside the expected range for an individual patient and adequate scan quality has been confirmed, this should prompt consideration for a re-evaluation of the patient and plan of care.
- A DXA report (baseline and follow-up) should state that a follow-up exam is recommended as long as a valid comparison is available, and the precise timing depends particular clinical circumstances.
- If the DXA interpreter has adequate clinical information, a precise timing for the next BMD should be recommended; otherwise, a general recommendation about repeat testing should still be part of the report.

## Part V: Summary and future directions

DXA is widely used to diagnose osteoporosis, assess fracture risk, and monitor changes in BMD. The clinical utility of DXA is highly dependent on the quality of the scan acquisition, analysis, and interpretation. Healthcare professionals are best equipped to manage patients when BMD measurements are performed accurately and precisely and when interpretation follows well-established standards. VFA, when feasible, can be used in selected patients in whom a DXA is performed. The strengths of DXA include its wide availability, well-validated system including large normative databases, ease of performance and very low radiation dose. DXA services should also regularly audit their practice and performance.

Standard operating procedures (SOPs) should be established for each DXA facility, with team training that includes physicians and technologists, to optimize DXA quality and reduce the error rate of DXA measurements [102]. In the future, there may be additional applications of DXA beyond those described here. Specifically, TBS is used as a determinant of bone “quality” to complement quantitative measurements. There are emerging periprosthetic and orthopedic uses of DXA [103], atypical femur fractures (AFF) assessment tools [59] and bone strain index (BSI) parameters that may soon have clinical applicability [104]. Hip axis length (HAL) can be assessed with DXA and may be associated with hip fracture risk in postmenopausal women [4]. Finally, DXA-based 3D modelling is a new technology to assess the trabecular and cortical bone compartments of the hip and may contribute to the monitoring of therapy [105, 106]. All these developments are of interest to our imaging field, but more evidence is needed to make recommendations for the application of these novel imaging techniques in clinical practice, e.g. for whole body composition with DXA.

Facilities that are needed but are not available for clinicians should be listed on a research agenda and proposed to our DXA community. The launch of a world-wide survey of the DXA community, to provide information about the distribution and types of DXA machines, including training and certification on DXA and protocols followed would be of interest as well, and how well the DXA vensors are included for the installation, cross-calibration, quality control and also technical and clinical education on the use of the new DXA system.

In conclusion, this updated DXA practice guideline provides recommendations to assist imaging specialists and clinicians in requesting, performing and interpreting DXA and VFA. The updated DXA practice guideline did not include applications in pediatrics, but will be followed in another publication of IWG.

### *Statements based on IWG consensus:*

- DXA BMD measurement should be performed at the lumbar spine, total hip, femoral neck and, if indicated one-third radius.
- Consider DXA in all women at the age ≥ 65 years, men age > 70 years, and women and men age ≥ 50 years with risk factors for osteoporosis (Table 4).
- Evaluate for prevalent vertebral fractures with VFA or standard radiography in patients ≥ 50 years with specific risk factors, or with a T-score < -1.0 in older men and women, historical height loss > 4 cm, self-reported but undocumented vertebral fracture, or long-term glucocorticoid therapy (Table 5).
- Consider DXA in younger adults (premenopausal women and men under 50 years) with specific diseases, and/or medical drugs and/or fracture.
- Each DXA facility should determine its precision error and calculate the least significant change (LSC), to be repeated when a new DXA system is installed.
- In accordance with the established WHO operational definition, osteoporosis is diagnosed based on a T-score of − 2.5 or lower in the lumbar spine, femoral neck, total hip, or one-third radius. The lowest T-score at any of these measured sites should be used for diagnosis.
- Some societies presumed a diagnosis of osteoporosis in the presence of low-trauma major fracture (hip, spine, forearm, humerus, pelvis).
- The NHANES III reference database is recommended for T-score calculation and depending on the society based on 20–29 years aged White women or same sex-type.
- Recommend follow-up DXA as indicated, depending on clinical circumstances.
- Follow-up of patients should ideally be conducted in the same facility with the same DXA system, if the acquisition, analysis, and interpretation adhere to recommended standards.
- The frequency of BMD testing in clinical practice may be influenced by the patient’s clinical state, national clinical guidelines, cost and reimbursement. Suggested intervals between BMD testing are typically 1–5 years after starting or changing therapy.
- Procedural certification and repeated audits are recommended.

#### EANM Liability statement

“This guideline summarizes the views of the European Association of Nuclear Medicine (EANM), American Association of Clinical Endocrinology (AACE), American Society for Bone and Mineral Research (ASBMR), Asian Federation of Osteoporosis Societies (AFOS), Canadian Society of Endocrinology and Metabolism (CSEM), Canadian Association of Nuclear Medicine (CANM), European Calcified Tissue Society (ECTS), European Society for Clinical and Economic Aspects of Osteoporosis, Osteoarthritis and Musculoskeletal Diseases (ESCEO), ), European Society of Radiology (ESR), European Society of Musculoskeletal Radiology (ESSR), International Osteoporosis Foundation (IOF), International Society for Clinical Densitometry (ISCD), Korean Society of Bone and Mineral Research (KSBMR), and the Radiological Society of North America (RSNA).

The recommendations should be taken into context of good practice of nuclear medicine and do not substitute for national and international legal or regulatory provisions”.

## Data Availability

Data sharing not applicable to this article as no datasets were generated or analysed during the current study.
